# Manipulation of *p*-/*n*-Type Thermoelectric Thin Films through a Layer-by-Layer Assembled Carbonaceous Multilayer Structure

**DOI:** 10.3390/mi9120628

**Published:** 2018-11-28

**Authors:** Wonjun Jang, Hyun A Cho, Kyungwho Choi, Yong Tae Park

**Affiliations:** 1 Department of Mechanical Engineering, Myongji University, Yongin, Gyeonggi 17058, Korea; wjang@mju.ac.kr (W.J.); chocho9508@hanmail.net (H.A.C.); 2 New Transportation Innovative Research Center, Korea Railroad Research Institute, 176, Cheoldobangmulgwan-ro, Uiwang-si, Gyeonggi-do 16105, Korea

**Keywords:** thermoelectric, layer-by-layer, graphene, carbon nanotube, thin film

## Abstract

Recently, with the miniaturization of electronic devices, problems with regard to the size and capacity of batteries have arisen. Energy harvesting is receiving significant attention to solve these problems. In particular, the thermoelectric generator (TEG) is being studied for its ability to harvest waste heat energy. However, studies on organic TEGs conducted thus far have mostly used conductive polymers, making the application range of TEGs relatively narrow. In this study, we fabricated organic TEGs using carbonaceous nanomaterials (i.e., graphene nanoplatelet (GNP) and single-walled carbon nanotube (SWNT)) with polyelectrolytes (i.e., poly(vinyl alcohol) (PVA) and poly (diallyldimethyl ammonium chloride) (PDDA)) via layer-by-layer (LbL) coating on polymeric substrates. The thermoelectric performance of the carbonaceous multilayer structure was measured, and it was confirmed that the thermoelectric performance of the TEG in this study was not significantly different from that of the existing organic TEG fabricated using the conductive polymers. The 10 bilayer SWNT thin films with polyelectrolyte exhibited a thermopower of −14 μV·K^−1^ and a power factor of 25 μW·m^−1^K^−2^. Moreover, by simply changing the electrolyte, *p*- or *n*-type TEGs could be easily fabricated with carbonaceous nanomaterials via the LbL process. Also, by just changing the electrolyte, *p*- or *n*-type of TEGs could be easily fabricated with carbonaceous nanomaterials with a layer-by-layer process.

## 1. Introduction

The internet of things (IoT) and wearable devices market is experiencing rapid growth. As a result, the demand for batteries to be used in these devices is increasing; however, increasing miniaturization of electronic devices is limited by the replacement of batteries and explosion risk [1,2]. To solve these problems, various approaches have been studied, among which energy harvesting is currently the most significant.

Energy can be harvested by triboelectric [3], thermoelectric [4], or piezoelectric [5] techniques, and extensive thermoelectric research is underway owing to the advantages of harvesting waste heat. The thermoelectric generator (TEG) harvests electricity via the Seebeck effect, which states that thermoelectric power is generated when there is a temperature gradient in the system. Traditional TEGs have been fabricated using inorganic semiconductors such as bismuth telluride to maximize the Seebeck effect [6,7,8]. However, the conventional thermoelectric materials possess the disadvantages of being hard, deformable, toxic, and expensive. Therefore, studies regarding organic thermoelectric materials have been actively conducted to overcome these limitations [9,10]. An organic TEG is basically composed of conductive polymers such as polyaniline (PANi) or poly(3,4-ethylenedioxythiophene) doped with poly(styrene sulfonate) anions (PEDOT:PSS). It has a low thermal conductivity and can be deposited on a flexible substrate, indicating that conductive materials are advantageous thermoelectric materials. Moreover, the incorporation of carbon-based nanofillers in the polymer matrix can enhance or manipulate the electrical properties of the nanocomposites. Therefore, studies have been focused on enhancing the thermoelectric effect using composites with carbonaceous nanomaterials such as graphene or carbon nanotubes (CNTs) [11,12,13,14,15,16]. Several methods exist to fabricate polymeric composites with carbonaceous nanomaterials. In this study, polymer-CNT and polymer-graphene multilayers were fabricated using a simple, inexpensive, and versatile method.

The layer-by-layer (LbL) technique, which has been studied for a long time as one of the wet coating methods, has gained attention as a very simple and low-cost process [17,18]. In particular, it is possible to fabricate flexible thin films of various materials on a substrate, such as cotton, which is difficult to coat using conventional methods. Using the LbL method, various properties such as flame retardancy [19], electrical conductivity [20], or sensor characteristics [21], can be imparted to the desired materials. However, only a few papers have observed the thermoelectric properties of the LbL-assembled thin film TEGs [22,23,24,25]. In this study, the thermoelectric properties of graphene or CNT thin films coated on a poly(ethylene terephthalate) (PET) substrate were analyzed.

Carbonaceous nanomaterials with a high electrical conductivity, such as graphene and CNTs, have been studied with organic/inorganic composite TEGs combined with polymers. Since these studies aim to increase the thermoelectric performance of an organic TEG, conductive polymers (e.g., PANi, PEDOT:PSS, etc.) were mostly used for organic materials. In this study, the thermoelectric properties of the TEG were investigated using poly(vinyl alcohol) (PVA) and poly(diallyldimethyl ammonium chloride) (PDDA) without a conducting polymer. In addition, using the LbL technique, it was possible to readily fabricate a carbonaceous nanomaterial-polymer composite TEG, enabling the wider use of organic TEGs.

## 2. Materials and Methods Methods

### 2.1. Materials

**Figure 1:**
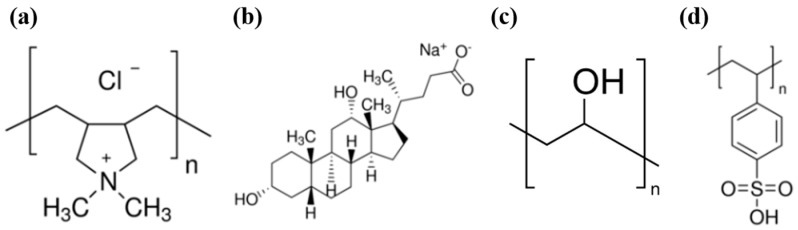
Chemical structures of (**a**) poly(diallyldimethyl ammonium chloride) (PDDA), (**b**) sodium deoxycholate (DOC), (**c**) poly(vinyl alcohol) (PVA), and (**d**) poly(4-styrenesulfonic acid) (PSS).

Purified electric arc single-walled carbon nanotubes (SWNTs, TUBALL™ SWNT, individual tube: average 1 μm length and 2 nm diameter, carbon content 75%) and graphene nanoplatelets (GNPs, Angstron, maximum X–Y dimensions of 10 μm, carbon content 95%, oxygen content ≤ 2.5%) were used in this study. PDDA (M*_w_*~200,000–350,000 g/mol, Figure 1a), sodium deoxycholate (DOC, C_24_H_39_NaO_4_, Figure 1b), PVA (M*_w_*~89,000–98,000 g/mol, Figure 1c), poly(4-styrenesulfonic acid) (PSS, M*_w_*~75,000 g/mol, 18 wt% in H_2_O, Figure 1d), isopropyl alcohol (IPA), and methanol were purchased from Sigma-Aldrich (Yongin, Korea). All chemicals were used as received. A PET film (100 μm thickness, Goodfellow, Huntingdon, England, UK) and single-side-polished silicon wafers (University Wafer, Boston, MA, USA) were purchased as substrates.

### 2.2. Layer-by-Layer Assembly

**Figure 2:**
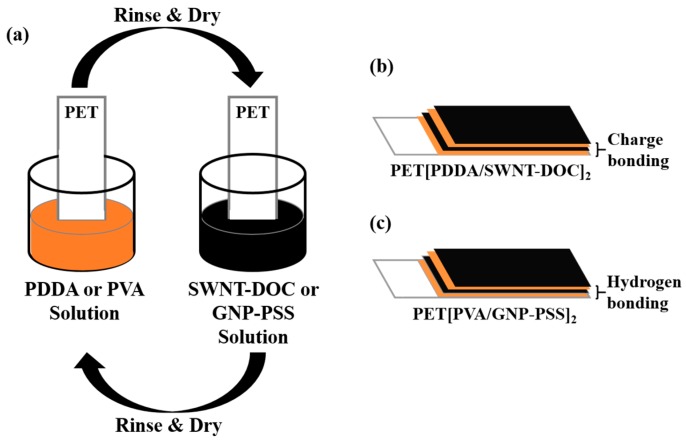
(**a**) Schematics of layer-by-layer process for PET[PDDA/SWNT-DOC] and PET[PVA/GNP-PSS] thin films. (**b**) Schematics of a PET[PDDA/SWNT-DOC]_2_ sample based on charge bonding. (**c**) Schematics of a PET[PVA/GNP-PSS]_2_ sample based on hydrogen bonding.

The LbL technique was used in this study to uniformly coat GNPs or SWNTs on the PET substrate (see Figure 2). First, 0.05 wt% SWNTs were added to deionized (DI) water with 1.5 wt% DOC and dispersed for 60 min using a tip sonicator (UW2070, Banderin Electronic, Berlin, Germany). CNTs require special chemicals to disperse in water owing to their entanglement and hydrophobicity. DOC is widely known as a surfactant that aids in the proper dispersion of SWNTs in deionised (DI) water [26]. The prepared SWNT-DOC solution had a negative charge [27]. Subsequently, 0.25 wt% PDDA was added to DI water and dispersed for 30 min using a bath sonicator (Branson, CPX-3800H, Emerson Electric Company, Ferguson, MO, USA). The PDDA solution had a positive charge, which serves as a counterpart of the SWNT-DOC solution. The PET film, which was surface-cleaned using a plasma etcher (Harrick Plasma, PDC 32G-2, Ithaca, NY, USA) for 5 min, was initially applied to PDDA, a positively charged solution, for 5 min. After rinsing and drying, it was placed in the SWNT-DOC solution, which was a negatively charged solution. After 5 min, rinsing and drying were repeated, by which the PDDA and SWNT-DOC layers were uniformly deposited on the PET substrate by charge bonding [20,27]. One bilayer (BL) was generated by the first cycle and consisted of one layer of PDDA and SWNT-DOC. Thus, in the case of *n* BL coating, PDDA and SWNT-DOC multilayers were coated on the PET substrate, and this was labeled as PET[PDDA/SWNT-DOC]*_n_*. Similarly, the GNP multilayers were coated on a PET substrate as follows: 0.1 wt% GNP was added to DI water with 0.1 wt% PSS and dispersed for 180 min using a tip sonicator. PSS is widely known as a dispersing agent to help disperse GNP properly in DI water. This is because the sulfonic functional groups of PSS prevent the aggregation of GNP in DI water [28]. As a counterpart of the GNP-PSS solution, 0.25 wt% PVA was added to DI water and dispersed via magnetic stirring at 70 °C for 30 min. PVA forms a hydrogen bond with PSS, allowing the stable formation of multilayers of GNP-PSS and PVA [28,29]. Similarly, each layer of PVA and GNP-PSS was labeled as 1 BL and a PVA and GNP-PSS multilayer film coated on the PET substrate was labeled as PET[PVA/GNP-PSS]*_n_*.

### 2.3. Characterization

LbL thin films on a PET substrate were used to analyze UV–Visible (UV–Vis, Ocean optics, Largo, FL, USA) light absorbance. The thicknesses of the LbL films deposited on the thermally oxidized silicon wafers were determined using a spectroscopic ellipsometer (SE, V-VASE, J.A. Woollam Co., Lincoln, NE, USA). Surface images of the LbL-coated PET samples were observed using a field emission scanning electron microscope (FE-SEM) device (SU-70, Hitachi, Tokyo, Japan) at 15 kV. Images for cross-sections of both samples were obtained using a transmission electron microscope (TEM, JEM-2100F, JEOL, Tokyo, Japan) to characterize the carbonaceous nanomaterial-polymer multilayers on PET.

For thermoelectric performance measurement, a PET[PVA/GNP-PSS] or PET[PDDA/SWNT-DOC] LbL sample (width: 10 mm, length: 55–60 mm) was installed between two Peltier devices (Marlow industries, Dallas, TX, USA) acting in opposite directions. The Peltier device initiates thermoelectric measurement by providing a temperature gradient to the test sample. The temperature gradient of the sample can be measured with a thermocouple composed of copper and constantan. The Peltier device operates in the temperature range of −4 to +4 K, and the maximum temperature difference range is approximately 7 K. Subsequently, to measure the electrical resistance and voltage of the specimen, a silver paste was applied to the surface of the specimen to minimize the electrical contact resistance. Thereafter, with a four-point probe, the electrical resistance was measured using a pair of electrodes, and the potential difference was measured by connecting a voltmeter to the other pair. The electrical resistance was obtained from the I-V curve, and the thermopower was obtained from the V-T curve.

## 3. Results

**Figure 3:**
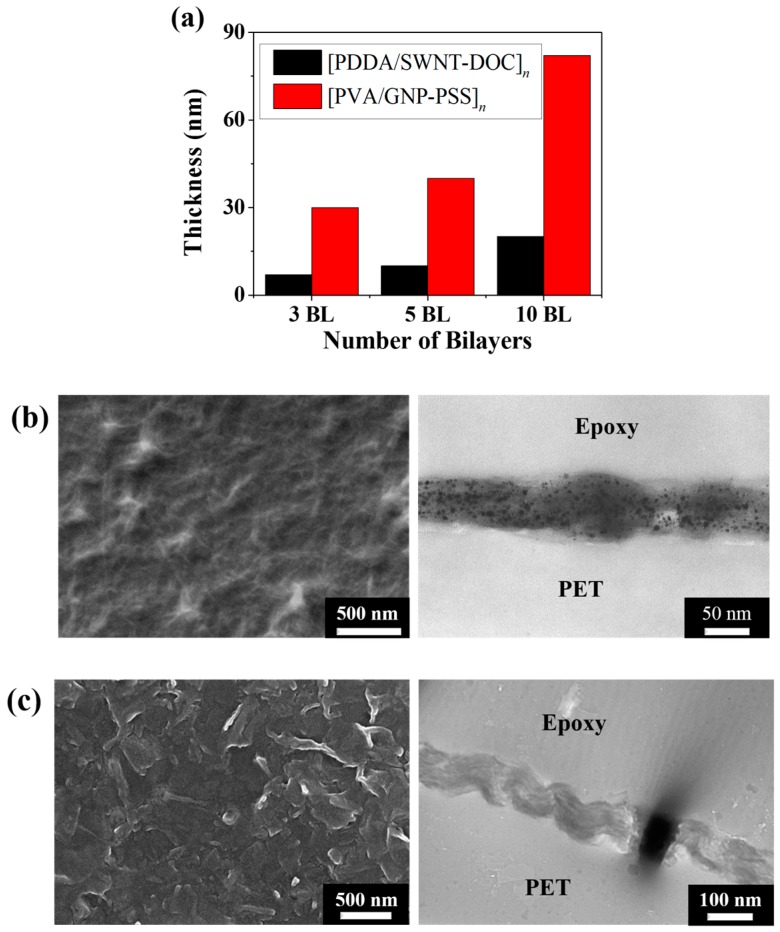
(**a**) Ellipsometric thickness of [PDDA/SWNT-DOC]*_n_* and [PVA/GNP-PSS]*_n_* (*n* = 3, 5, and 10) thin films. Both increase uniformly as the number of BLs increases. SEM surface images and TEM cross-sectional images of (**b**) [PDDA/SWNT-DOC]_20_ and (**c**) [PVA/GNP-PSS]_20_ thin films.

Figure 3a shows the thickness of the [PDDA/SWNT-DOC] and [PVA/GNP-PSS] LbL thin films. The thickness increases with an increase in the number of BLs in both systems. SWNT LbL thin films, with a thickness of approximately 7 nm at 3 BLs, increase in thickness to 20 nm at 10 BLs. This suggests that SWNT is linearly coated as the number of BLs increases. The GNP LbL thin film possesses a thickness of 30 nm at 3 BLs, which increases to 82 nm at 10 BLs. It also demonstrates that GNPs are linearly coated with increasing BLs. The SWNT LbL film is thinner than the GNP sample, because GNP is a 2-D platelet and SWNT is a 1-D tube [27,29]. As the number of BLs increases, the GNP LbL thin film becomes relatively thicker than the SWNT film, as shown in the SEM and TEM images in Figure 3b,c.

**Figure 4:**
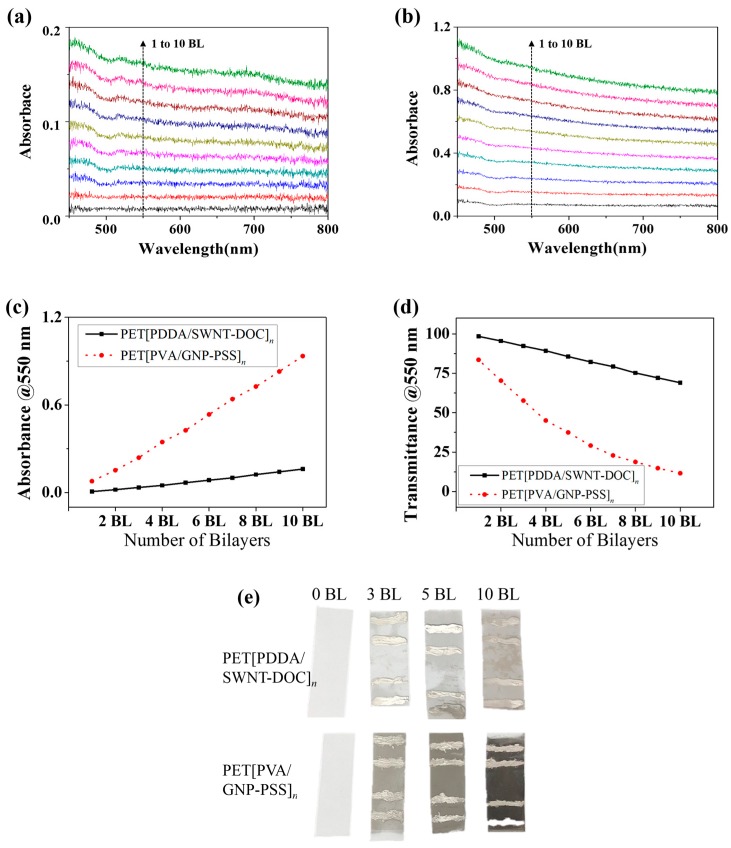
(**a**) Visible light absorbance spectra of PET[PDDA/SWNT-DOC]*_n_* (*n* = 1–10) LbL thin films. (**b**) Visible light absorbance spectra of PET[PVA/GNP-PSS]*_n_* (*n* = 1–10) LbL thin films. (**c**) Visible light absorbance of PET[PDDA/SWNT-DOC]*_n_* (*n* = 1–10) (solid line) and PET[PVA/GNP-PSS]*_n_* (*n* = 1–10) (dotted line) at 550 nm wavelength. (**d**) Visible light transmittance of PET[PDDA/SWNT-DOC]*_n_* (*n* = 1–10) (solid line) and PET[PVA/GNP-PSS]*_n_* (*n* = 1–10) (dotted line) at 550 nm wavelength. (**e**) Images of PET[PDDA/SWNT-DOC]*_n_* (*n* = 3, 5, 10) and PET[PVA/GNP-PSS]*_n_* (*n* = 3, 5, 10) with control (0 BL) samples. In the LbL samples above, the silver material on each sample surface is silver paste applied for four-point probe measurement.

Figure 4a,b show the light absorbance of PET[PDDA/SWNT-DOC] and PET[PVA/GNP-PSS] with an increasing number of BLs. As shown in Figure 4c, the light absorbance increases uniformly from 1 to 10 BLs in both thin films. This indicates that SWNT and GNP layers are linearly deposited during LbL coating. In addition, it can be confirmed that SWNT bilayers exhibit a relatively much lower absorbance than GNP because SWNT is a 1-D material with an exceptionally small diameter and GNP is a relatively larger 2-D platelet. Based on its characteristics, SWNT is widely used as a transparent electrode in several studies [30,31,32]. Figure 4c,d show the absorbance and transmittance at 550 nm in the two LbL systems. As can be observed from Figure 4c, the absorbance linearly increases to 0.16 for PET[PDA/SWNT-DOC]_10_ and to 0.9 for PET[PVA/GNP-PSS]_10_. Conversely, the light transmittance decreases to 69% for PET[PDA/SWNT-DOC]_10_ and 11.6% for PET[PVA/GNP-PSS]_10_. This can be observed more clearly in Figure 4e, which shows 3, 5, and 10 BL PET[PDA/SWNT-DOC] and PET[PVA/GNP-PSS] samples with a bare PET (0 BL), respectively. Both carbonaceous nanomaterials (i.e., SWNT and GNP) demonstrate that as the number of BLs increases, the color of the LbL thin films gradually darkens; in particular, the GNP LbL thin film is distinctly darker.

**Figure 5:**
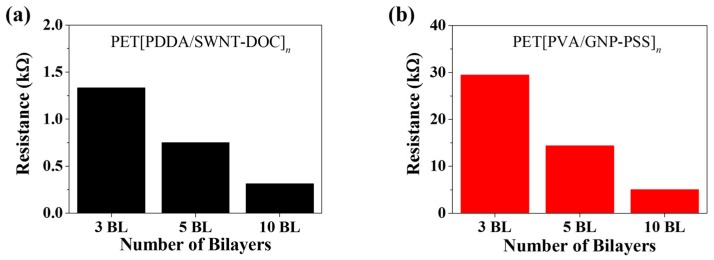

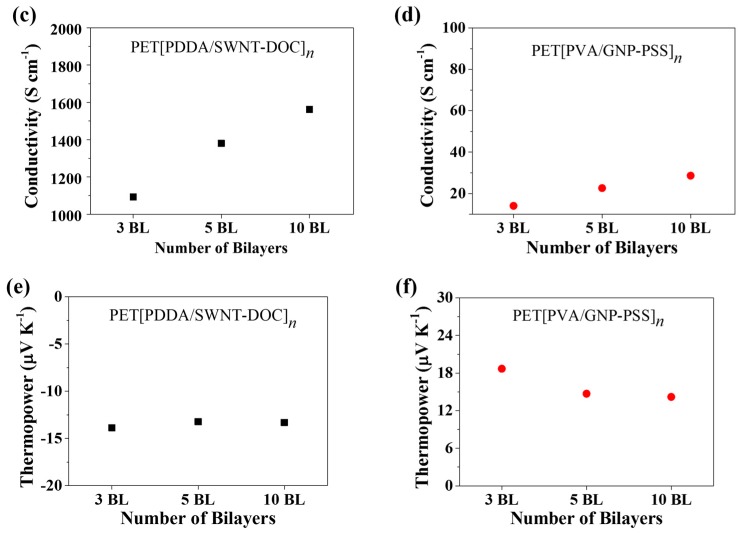
(**a**,**b**) Electrical resistance of PET[PDDA/SWNT-DOC]*_n_* (*n* = 3, 5, 10) and PET[PVA/GNP-PSS]*_n_* (*n* = 3, 5, 10) LbL thin films. (**c**,**d**) Electrical conductivity of PET[PDDA/SWNT-DOC]*_n_* (*n* = 3, 5, 10) and PET[PVA/GNP-PSS]*_n_* (*n* = 3, 5, 10) LbL thin films. (**e**,**f**) Thermopower (Seebeck coefficient) of PET[PDDA/SWNT-DOC]*_n_* (*n* = 3, 5, 10) and PET[PVA/GNP-PSS]*_n_* (*n* = 3, 5, 10) LbL thin films.

Figure 5a shows the electrical resistance of PET[PDDA/SWNT-DOC] LbL thin films at 3, 5, and 10 BLs. As a result of the ellipsometric thickness and light absorbance, the amount of SWNT coated on the PET increases as the number of BLs increases, so that the absorbance and thickness also increase. Likewise, the electrical resistance decreases as the number of BLs deposited increases. The electrical resistance of PET[PDDA/SWNT-DOC] thin films decreases from 1.3 kΩ to 0.31 kΩ at 3 to 10 BLs, respectively. Similarly, Figure 5b also shows the decrease in the electrical resistance of PET[PVA/GNP-PSS] thin films according to the increase in the number of BLs deposited. The electrical resistance decreases from 29.5 kΩ to 5.0 kΩ at 3 to 10 BLs, respectively. In this case, it can be confirmed that the electrical resistance of the SWNT LbL thin film is much lower than that of the GNP LbL thin film, because the SWNT can form a 3-D network with an increasing number of deposited BLs and can enable considerably efficient electron transport. This can be more distinctly observed from the previous SEM and TEM images. Further, the GNP LbL thin film exhibits a relatively higher electrical resistance because it is stacked in a 2-D shape [3,29,33]. Figure 5c,d show the electrical conductivity obtained by applying geometrical factors to the measured resistance values. GNP LbL thin films with a relatively increased thickness and a 2-D network structure adversely affect the carrier transport, demonstrating a conductivity of approximately 10 S·cm^−1^, but the SWNT samples demonstrated an increase of 2 orders. In both samples, the conductivity increased with increasing number of BLs owing to the formation of more pathways for electrical carriers. Figure 5e,f show the thermopower values of SWNT and GNP LbL thin films, respectively. For SWNT and GNP, the values of thermopower are negative and positive, respectively. This is because the PDDA/SWNT-DOC multilayers are *n*-type thermoelectric materials with electrons being the major carrier and PVA/GNP-PSS multilayers are *p*-type thermoelectric materials. SWNT LbL thin films have a constant thermopower value of approximately −14 μV·K^−1^, whereas that of GNP is approximately 15 μV·K^−1^. This is an interesting result because a majority of the *n*-type organic nanocomposites demonstrate a relatively low conductivity compared to *p*-type composites due to the conversion of the major carrier from holes to electrons. Free-standing organic nanocomposites with PEI-doped SWNT exhibit 1–10 S·cm^−1^ [34] and LbL multilayers of a PEI-doped double-walled carbon nanotubes composite demonstrate up to 300 S·cm^−1^ of conductivity with 80 BLs [35]. In this research, an electrical conductivity of 10^3^ S·cm^−1^ was achieved through only 3 to 10 BLs of SWNTs with a negative thermopower of ~15 μV·K^−1^. Although the doping mechanism and carrier transportation process is beyond this research, it is valuable to report this result since a high current output in the thermoelectric generation system is a problem in industrial applications of organic thermoelectric materials.

**Figure 6:**
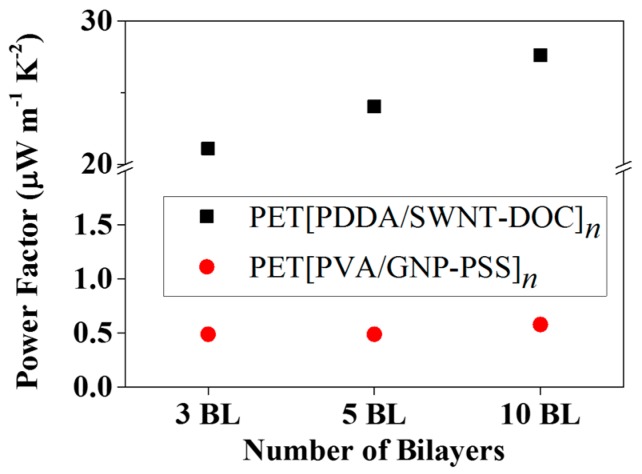
Thermoelectric power factor of PET[PDDA/SWNT-DOC]*_n_* (*n* = 3, 5, 10) and PET[PVA/GNP-PSS]*_n_* (*n* = 3, 5, 10) LbL thin films.

In Figure 6, with the measured electrical conductivity and thermopower, the power factor (P.F. = S^2^·*σ*) was calculated as a function of the number of BLs. Although the absolute values of the thermopower of both SWNT and GNP LbL thin films are similar, the power factor of the 10 BL SWNT sample was 28 μW·m^−1^K^2^, whereas that of the GNP sample was less than 1 μW·m^−1^K^2^. This is comparable to other carbonaceous organic nanocomposites with an exceedingly high conductivity, even among *p*-type organic thermoelectric materials [36,37,38].

## 4. Conclusions

Carbonaceous nanomaterial-polymer multilayer composites (i.e., [PDDA/SWNT-DOC]*_n_* and [PVA/GNP-PSS]*_n_*) were applied to fabricate cost-effective, scalable, and flexible TEGs, where high performance and high versatility (*p*- and *n*-type TEGs) were demonstrated. Alternate exposure of the PET substrate to two opposite aqueous solutions yielded LbL thin films with a linear trend of growth, evidenced by the thickness and light absorbance. [PDDA/(SWNT-DOC)]_10_ films possess a conductivity, thermopower, and power factor of 1.8 × 10^3^ S·cm^−1^, −14 μV·K^−1^ (*n*-type thermoelectric material), and 28 μW·m^−1^K^2^, respectively. Compared with previous organic/inorganic composite TEGs fabricated using conventional conductive polymers, the thermopower of the LbL thin films in this study was very similar. The SWNT-based thin film was a flexible TEG, and is capable of replacing conventional brittle TEGs in certain energy harvesting applications. Moreover, the carbonaceous nanomaterial-polymer composite TEG is expected to lower the cost of TEG fabrication and enable fabrication using a wider variety of materials. Further study of other types of LbL materials (i.e., polymers, dispersions, etc.) would further reduce the electrical resistance and increase the thermopower of thin-film TEGs.
